# From Cellulose to Cellulose Nanofibrils—A Comprehensive Review of the Preparation and Modification of Cellulose Nanofibrils

**DOI:** 10.3390/ma13225062

**Published:** 2020-11-10

**Authors:** Tan Yi, Hanyu Zhao, Qi Mo, Donglei Pan, Yang Liu, Lijie Huang, Hao Xu, Bao Hu, Hainong Song

**Affiliations:** 1 College of Light Industry and Food Engineering, Guangxi University, Junwu Rd, Xixiangtang District, Nanning 530004, China; yitangxu@st.gxu.edu.cn (T.Y.); 1816301036@st.gxu.edu.cn (H.Z.); 1705170129@st.gxu.edu.cn (Q.M.); 1705160234@st.gxu.edu.cn (D.P.); jiely165@gxu.edu.cn (L.H.); 1705170121@st.gxu.edu.cn (H.X.); 1705170222@st.gxu.edu.cn (B.H.); 2 Guangxi Key Laboratory of Clean Pulp & Papermaking and Pollution Control, Junwu Rd, Xixiangtang District, Nanning 530004, China; 3 Guangxi Bossco Environmental Protection Technology Co., Ltd., 12 Kexing Road, High-tech Zone, Nanning 530012, China; songhn@bossco.cc

**Keywords:** cellulose nanofibrils, preparation process, surface modification, chemical modification

## Abstract

This review summarizes the preparation methods of cellulose nanofibrils (CNFs) and the progress in the research pertaining to their surface modification. Moreover, the preparation and surface modification of nanocellulose were comprehensively introduced based on the existing literature. The review focuses on the mechanical treatment of cellulose, the surface modification of fibrillated fibers during pretreatment, the surface modification of nanocellulose and the modification of CNFs and their functional application. In the past five years, research on cellulose nanofibrils has progressed with developments in nanomaterials research technology. The number of papers on nanocellulose alone has increased by six times. However, owing to its high energy consumption, high cost and challenging industrial production, the applications of nanocellulose remain limited. In addition, although nanofibrils exhibit strong biocompatibility and barrier and mechanical properties, their high hydrophilicity limits their practical application. Current research on cellulose nanofibrils has mainly focused on the industrial production of CNFs, their pretreatment and functional modification and their compatibility with other biomass materials. In the future, with the rapid development of modern science and technology, the demand for biodegradable biomass materials will continue to increase. Furthermore, research on bio-based nanomaterials is expected to advance in the direction of functionalization and popularization.

## 1. Introduction

**Figure 1:**
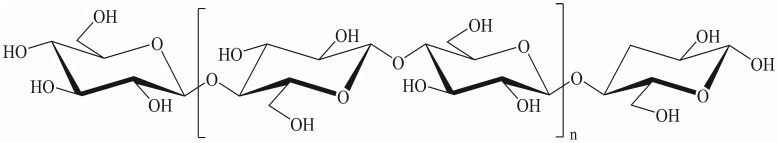
Cellulose molecular structure.

As the most abundant natural polymer on earth, cellulose occupies 40–50% of the earth’s total biomass reserves. The cellulose solely produced through photosynthesis is as high as 10^11^–10^12^ tons per year [1,2] and has always been regarded as an inexhaustible green resource [3]. In the natural environment, cellulose is mainly stored in plants and microorganisms [3]. Its molecular structure is shown in Figure 1. The molecular backbone of cellulose is a linear rigid chain linked by β-D-glucopyranose (AGU) through 1,4-glycosidic bonds in a chair-shaped conformation [4]. The presence of tri-and hydroxyl groups, resulting from the strong hydrogen bonds composed of polyhydroxyl groups, indicate that the cellulose has a high cohesive microfiber network structure and the hydrogen bonding effect makes cellulose difficult to dissolve in common solvents [1,5,6].

In 1982, Turbak et al. used a high-pressure homogenizer to extract nanocellulose, namely cellulose nanofibrils (CNFs), from eucalyptus pulp [3,7]. With advancing research, CNFs have exhibited high transparency, excellent mechanical properties and good biocompatibility. They have been gradually applied to antibacterial packaging, corrosion inhibitor carriers, gels, transparent conductive films, 3D printing, composite materials and drug delivery [8,9,10]. At the same time, compared with other nanocellulose fibers, CNFs have a simple preparation process. CNFs were developed earlier and are currently the main target for the industrial production of nanocellulose. This review focuses on nanocellulose fibrils [1,11,12].

Although CNFs demonstrate excellent functionality, they have not yet been industrialized. The industrial production of CNFs has been mainly hindered by two factors. Firstly, CNFs are primarily manufactured by high-pressure homogenizers, high-energy ball mills (mechanical chemistry), microfluidizers, ultra-low temperature crushing and other methods. However, because of the relatively high lengths and diameters of CNFs, their specific surface area is large, the surface hydroxyl groups easily form hydrogen bonds and the fibers easily form hard agglomerations and are difficult to disperse. Simply relying on mechanical force to shear and separate the fibers generates a lot of energy consumption. In addition, the instantaneous high temperature generated during mechanical grinding affects the CNFs’ crystal structure, destroys their network structure and changes the gel behavior of the nanofibrils. Secondly, the fibrillation of cellulose must be completed in liquid and, after drying, irreversible hydrogen bonding occurs between the CNFsc fibers, that is, keratinization [13,14]. Therefore, CNFs products mostly exist as water dispersions with low solid contents and their storage space is large and their transportation cost is high. To make the preparation of CNFs easier, reduce the energy consumption required to prepare CNFs and obtain CNFs with a certain functional structure, a “two-step method” can be used for the preparation of nanofibrils. In this technique, the cellulose is pretreated first and then the microfibril structure of cellulose is peeled off by a gentle mechanical method to obtain the nanofibrils [11]. Among them, the pretreatment of cellulose involves three main techniques. (i) The use of cellulase hydrolysis to cut and peel off the fibers and make subsequent mechanical separation easier [15,16,17,18]. (ii) Chemical modification of cellulose, such as using coupling agents (lauric acid, malic acid, etc.) to replace the hydroxyl groups on the cellulose surface or by reacting with hydroxyl groups to reduce the hydroxyl content on the cellulose surface, thereby reducing the inter-cellulose and fiber. The hydrogen bond content and internal strength of the element make it easier to separate the fibers [19,20,21]. (iii) Chemical modification of cellulose surface hydroxyl groups, such as—using TEMPO oxidant to oxidize surface hydroxyl groups into aldehyde groups or carboxyl groups; cationization of cellulose fibers through the introduction of positive and negative charges to make cellulose through electrophilic addition or affinity; nucleus addition to introduce specific groups or components to promote the swelling of cellulose in water; or the introduction of the same charge to make fibers repel each other to reduce the cohesion between fibers [22,23,24,25,26]. Although the pretreatment of cellulose improves the ability of cellulose fibrillation, it also introduces functionalized structures or functional groups on the surface of cellulose and the levels of various factors in the experiment interact and are difficult to control, causing poor quality uniformity in the CNFs and challenging product evaluation [27].

Although CNFs exhibit excellent physical and chemical properties and have been used in numerous fields, their high hydrophilicity and gel behavior in solution have always hindered their development. At present, the main solution to this problem is to chemically modify the produced CNFs, by introducing functional groups or polymer molecules to the CNFs surfaces by chemical grafting or physical adsorption, thus introducing brand-new functions such as semiconductor electrical properties, ultra-high mechanical strength and excellent hydrophobicity.

This review introduces the preparation processes involved in CNFs pretreatment processes, where the main preparation process focused on is mechanical force and summarizes the most common raw fiber materials. We also detail the CNFs modification process and modification effects to make the preparation technology of CNFs more intuitive. Finally, we also consider the influence of different raw fiber material composition and characteristics, compare pretreatment methods for CNFs polymerization, crystallinity (CI) and functional features and examine the industrial capacity of cellulose in this detailed report.

## 2. Cellulose Raw Materials and CNFs

Plant cellulose mainly exists in the cell walls of plant fibers. According to the order of the cell wall formation, the chemical composition and structure varies and can be divided into the intercellular layer cell wall (*M*), primary wall (*P*) and the secondary wall (*S*). The intercellular layer is composed of linked cells and acts as a buffer between the cell structure. It does not contain cellulose and is instead mainly composed of lignin, hemicellulose and pectic substance formation. The primary wall is mainly secreted by the cell protoplast and is composed of cellulose, hemicellulose and pectic substances, demonstrating good elasticity and plasticity. The secondary wall is secreted by the protoplast in the primary wall via deposit growth. Cells stop growing when the secondary wall is formed. The secondary wall is approximately 5–10 m thick and generally consists of three layers: the outer layer of the secondary wall (*S*_1_), the middle layer of the secondary wall (*S*_2_) and the inner layer of the secondary wall (*S*_3_). The cellulose raw materials used to extract CNFs can come from wood, seed fibers (cotton fiber, cotton lint, kapok, etc.), plant bast fibers (hemp, flax, abaca, etc.) and various herbs (bagasse, straw, bamboo fiber) [2]. The average degree of polymerization of wood cellulose is approximately 10,000, that of cotton fiber is slightly higher at approximately 15,000 and that of herbal cellulose is slightly lower. In terms of fiber length, bast fiber has the longest fiber length, which can reach 120–180 mm and herbaceous straw has the shortest fiber length at approximately 1–2 mm. The fiber length for wood is approximately 3–5 mm. For the cellulose content, the cellulose content of the seed fibers, such as cotton, is the highest, usually above 95%, while wood fibers originally contain more impurities such as lignin and ash. The fiber content and fiber length of softwoods are higher than hardwoods. Generally, cellulose in raw plant materials mainly exists in mature plant cells [28,29]. Owing to the different physiological functions of plant cells, the thick-walled cells (fibers) of plants are most commonly used in production to reduce fiber fragments in CNFs products. For example, they can be used to remove parenchyma cells, stone cells, reticular wall cells and other structures. Generally, the higher the ratio of raw fiber materials used to prepare CNFs, the slenderer and uniform the single fibers, the lower the content of miscellaneous cells and the better the strength and fibrillation degree of the CNFs. Therefore, high-quality nanofibers are mostly made of pure cotton fibers [30]. The physical characteristics of cellulose raw materials mainly used in the production of CNFs are shown in Table 1. 

**Figure 2:**
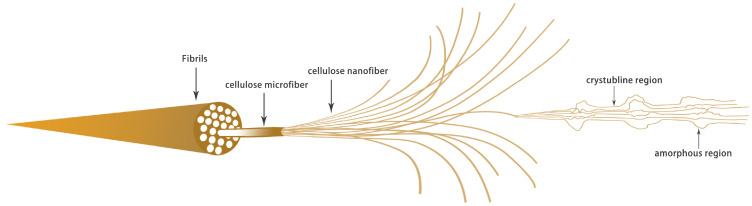
Internal structure of cellulose.

Nanocrystalline cellulose usually refers to tiny fibers with a diameter of less than 100 nm, which is the smallest physical structural unit in cellulose. Nanocrystalline cellulose can be divided into three types according to the different raw materials and processing methods: CNFs, crystalline nanocellulose (CNC) and bacterial nanocellulose (BNC) [34]. CNFs are cellulose prepared by mechanical methods or mild pretreatments for cellulose fibrillation. CNC makes use of the strong physical and chemical properties resulting from the removal of the cellulose amorphous area. Generally, the product CI of CNC is high its diameter is 5–70 nm, its length is 100–250 nm and its aspect ratio is low [35,36]. BNC is a kind of high-purity ribbon, like nanocellulose, which is synthesized by bacteria and other microorganisms, with a diameter of 20–100 nm and a length of several microns [37,38]. Generally, CNFs is a cellulose structure with a higher aspect ratio of 5–50 nm in diameter and several microns in length [39]; moreover, owing to the effect of high shear force in the production process, the fibers are entangled with each other to form a microfiber network structure, which easily forms a gel in aqueous solution [40]. Cellulose fiber is a kind of highly cohesive, multi-level structure, composed of a beta–1,4 glycosidic bond of a macromolecular, polysaccharide, rigid chain that, through intermolecular hydrogen bonding, intertwines to form a nanofiber by combining with a larger hemicellulose microfiber structure [41]. Figure 2 shows the fibrillation process from lignocellulose to cellulose nanofibrils and the crystal structure inside the cellulose nanofibrils.

## 3. Pretreatment of Cellulose

Enzymatic hydrolysis or chemical pretreatment of cellulose can effectively cut the cellulose and weaken the interaction of the secondary bonds between the fibers, thereby promoting the generation of nanocellulose during the preparation process and imparting new properties to the cellulose. However, the effect and site of each pretreatment process are different, along with the morphology of the fibrils produced. 

### 3.1. Cellulase Cutting Pretreatment

Cellulase has been widely used in the purification and saccharification of cellulose in the production of industrial ethanol, because it can cut cellulose macromolecules into small fragments. The production efficiency is high, green and environmentally friendly and the prepared CNFs products are high quality and widely used in CNFs pretreatments. Cellulase can be divided into C_1_ enzyme (endo-glucosidase), C_x_ enzyme (exo-glucosidase) and β-glucosidase. The enzymatic hydrolysis process can be roughly divided into three parts. (i) First, the C_1_ enzyme destroys the amorphous region in the cellulose chain. (ii) Second, the C_x_ enzyme then acts on the reduced end of the destroyed, non-directed region to decompose into β-1,4-glycosidic bonds. (iii) Finally, β-glucosidase decomposes the cellobiose and cellotriose produced in the first two parts into glucose molecules [42,43]. Cellulose enzymatic hydrolysis is different from acid treatment and its physical and chemical properties are relatively mild. By controlling the reaction conditions, CNFs with high aspect ratios can be obtained and the microfibril structure of nanofibrils can be retained [44,45]. 

As early as 2007, Pääkkö et al. [46] used a combination of enzymatic hydrolysis and high pressure homogenization to prepare CNFs with a high aspect ratio and network structure. After enzyme treatment, the filaments also demonstrated a rougher surface and the diameter gradually decreased from the intersection of the network structure to the center. In addition, CNFs under water suspension in the rheological test still exhibited appreciable gel properties. Henriksson et al. also used a similar method to prepare nanofibrils and compared them with the nanocellulose obtained after treatment with the same group of acids. It was evident from the experimental results that the CNFs prepared by enzymatic pretreatment and mechanical separation had a greater length than the nanocellulose. Compared with each other, the CNFs presented a network structure in which the chains were intertwined and overlapped. They demonstrated a higher viscosity in the water dispersion [47].

In addition, almost all studies have shown that the use of cellulase reduces the degree of polymerization (DP) of cellulose but increases the degree of CI. This is mainly attributed to the fact that exonuclease attacks the amorphous regions of cellulose. In addition, prolonged enzymatic hydrolysis destroys the cellulose crystallization area and causes a decrease in CI. In recent years, single-component studies on cellulase have also shown that endoglucose polymerase is the key to CNFs production. Nechyporchuk et al. [48] compared single-component endoglucanase (Fiber Care R) with cellulase (Celluclast 1.5 L) whose main activity was endoglucanase and exoglucanase. It was found that, compared with exoglucanase, endoglucanase demonstrated a weaker attack on the non-crystalline area and was better at dissociating the microfibrous structure of cellulose. The reduction played a limiting role (compared with a 30% reduction in the DP of the original cellulose) and at the same time reduced the content of low aspect ratio cellulose debris in the suspension (the fibril content was as high as 97%).

Due to the continuous accumulation of the cellulose microfibril structure during the enzymatic hydrolysis process, the effects of hemicellulose and lignin also further hinder the action of cellulase on the active site, causing the enzymatic hydrolysis rate to continue to decrease over time. To solve this problem, newly emerging cellulase and mixed cellulase systems have gradually been applied to the preparation of CNFs in recent years. Unlike traditional cellulase, most of these enzymes do not directly participate in the hydrolysis of cellulose but improve the contact between cellulase and cellulose fibers or improve the properties of fibrils without affecting the structure of cellulose, thereby promoting cellulase hydrolysis [49]. Penttilä et al. [50] found that xylan is an important factor affecting the rate of enzymatic hydrolysis and they used cellulase in a composite enzyme system composed of xylanase to prepare CNFs. Experiments found that xylanase can effectively prevent cellulose hydrolysis. The loose structure of the remaining cellulose during the process increases its CI from 43% to 48%. In addition, Long et al., through water retention value determination and Simons’ dyeing of cellulose, found that lysing polysaccharide monooxygenase and laccase can be used as auxiliary enzymes to selectively remove lignin and semi-fibrillated cellulose, which promotes the dissociation of cellulose so that cellulose can fully act on the hydrolysis site [51]. Bian et al. made use of a hybrid enzyme system composed of endoglucanase and xylanase, combined with mechanical grinding to prepare nanocellulose fibrinogen with an average diameter of only 15 nm and a water retention value up to 909% [52]. In addition, the report shows that the degree of fibrillation of cellulose treated with enzymes greatly increased, the content of lignin reduced and there were smaller water channels in the fiber network structure, which was the main reason for the high water content of the CNFs. 

Cellulose enzymatic hydrolysis is a simple, cheap, environmentally friendly and highly reproducible pretreatment process for CNFs preparation. Cellulase can promote the fibrillation of cellulose, which greatly increases the effect and efficiency of mechanical treatments. The use of a composite cellulase system also further improves the efficiency of this process, laying the foundation for the industrial production of CNFs. However, the disadvantage of enzymatic hydrolysis is that only functionalized single CNFs can be prepared and further modification is needed to expand their applications.

### 3.2. Chemical Pretreatment of Cellulose

Although enzymatic hydrolysis greatly improves the preparation efficiency of CNFs, the resulting products still have extremely high hydrophilicity and gel behavior. Moreover, each CNFs long chain is intertwined, owing to the action of hydrogen bonding; this greatly limits the application of cellulose in various fields. Different from enzymatic pretreatments, chemical modification of cellulose during cellulose pretreatments can provide cellulose with more functional characteristics. In general, chemical functional groups with specific functions in the pretreatment process mainly act on hydroxyl groups and pyranoid glucose rings on the cellulose surface. The introduction of functional groups leads to the substitution of hydroxyl groups between fibers and the introduction of charged groups, which weakens the cohesion between cellulose chains through repulsive forces, promotes the dispersion of nanocellulose in suspension and enables CNFs to have special electrical properties. Due to the osmotic pressure between cellulose and the dispersed liquid introduced by the functional group, water or other dispersed liquids can more easily penetrate the cellulose cell for cellulose swelling. Therefore, in practical applications, the pretreatment process combining enzymatic hydrolysis and chemical modification is often used. Generally, chemical modification pretreatment of cellulose includes anionization, cationization, introduction of aldehyde group and ring-opening of cellulose molecule. The anionization of cellulose mainly through carboxymethylation, phosphorylation, sulfonation and other ways to introduce electronegative groups to the surface hydroxyl of cellulose and cellulose has a better water solubility. On the contrary, cationization can be achieved by introducing cationic ammonium into the cellulose surface or by gas plasma treatment and cationic cellulose also has excellent water solubility and antistatic properties. The aldehyde group was introduced mainly through TEMPO oxidation or ozone oxidation. The ring-opening of cellulose was mainly treated by periodate oxidation.

#### 3.2.1. TEMPO Oxidation

2,2,6,6–tetramethylpiperidine–1–oxyl radical (TEMPO) is a piperidine nitroxide radical compound and TEMPO oxidation is a composite oxidation system including TEMPO, which is only weakly oxidizing. There are three main TEMPO co-oxidation systems: TEMPO-electrochemical oxidation, TEMPO-enzyme oxidation and TEMPO-co-oxidation systems [53,54]. Among these systems, TEMPO-electrochemical oxidation is a new and popular oxidation method, whereas TEMPO-co-oxidation system is simple to operate and has a high oxidation yield and is a relatively common oxidation method [55].In the presence of the TEMPO co-oxidation system, owing to the difference in reactivity, it can selectively oxidize only the primary hydroxyl groups on the cellulose. After the TEMPO oxidation of cellulose, carboxyl groups can be directly introduced into the cellulose molecule, thereby significantly improving the activity and adsorption performance of the cellulose, while reducing the agglomeration capacity of the cellulose. Through subsequent mechanical stratification and adjustment of the size of the gap between fibers, TEMPO oxidized nanocellulose (TOCNFs) is widely used in drug delivery [56]. As early as 2006, Tsuguyuki et al. [57] proposed the oxidization of cellulose with TEMPO and the introduction of aldehyde groups on the surface of cellulose. Aldehyde cellulose with an aldehyde group content of 0.225 mmol/g was obtained by adjusting the pH = 10.5, oxidizing TEMPO/NaBr (1:10) for 30 min and increasing the wet strength of the paper sheet to 4.9 Nm/g. It was proved that the aldehyde groups on the surface of cellulose formed hemiacetal bonds with the hydroxyl groups on the surface of cellulose. Many studies have also proved that TEMPO oxidized cellulose can produce various structures such as nanocellulose networks, nanofibrils and nanocellulose crystals by controlling the carboxylate content and processing technology [23]. When the cellulose carboxylate content is >1 mmol/g, a high-viscosity transparent gel can be obtained after gentle mechanical decomposition [58,59,60,61]. After treatment, the average length of a single fiber is 3 nm and the length is a few microns. When the cellulose is first processed after mechanical treatment followed by TEMPO oxidation, a translucent dispersion composed of nanofibril networks can be obtained. The product is similar to the CNFs obtained by enzymatic hydrolysis or without pretreatment [57,62]. However, when the TEMPO-CNFs water dispersion system was ultrasonically treated, a needle or short rod CNC could be obtained, in which the average width of the fiber was 3 nm and the average length was 150–200 nm [63,64]. Although the reaction time for preparing nanocellulose by TEMPO oxidation is short and the degree of cellulose functionalization is high, the cellulose aldehyde group is prone to various side reactions during the reaction, such as ring opening of the cellulose AGU and lowering the DP of cellulose. Additionally, the degree of the oxidation reaction is more difficult to control. In this regard, Saito et al. [65] used a TEMPO/NaClO/NaClO_2_ oxidation system to oxidize under neutral conditions to obtain an aldehyde-free CNFs with an average diameter of 5 nm, an average length of more than 2 µm and a DP of more than 900. When the carboxyl group content increased to 0.8 mmol/g, the yield of nanocellulose was as high as 90%. In 2011, Aracri et al. [66,67] reported a new type of laccase-TEMPO oxidation system, which effectively improved the wet strength of sisal cellulose. At the same time, the oxidation system can be oxidized in a more neutral environment to ensure the system selectively oxidizes cellulose while avoiding the use of harmful halides.

Recently, Alves et al. [68] reported including acetic acid, lactic acid, phosphoric acid, citric acid and so on many kinds of edible acid for TOCNFs water slurry rheological behavior and the effect of clustering, improved TOCNFs because of low polymerization degree, low degree of branching and not easy to form a gel under acid condition, under acid condition to build the reticular structure provides a theoretical basis.

Although TOCNFs demonstrates good performance and can complete the functionalization of cellulose or charge the fiber during pretreatment, the oxidation process of TEMPO is not direct and the reaction depends on the pH value. The reaction system is more toxic and pollutes the environment. The washing process is cumbersome, so there is still no industrial production. However, simplifying its reaction technology and making the reaction system recyclable will greatly increase the prospects for the industrial production of TOCNFs.

The current, most common TEMPO oxidation system compositions are shown in Table 2.

#### 3.2.2. Carboxymethylation

**Figure 3:**
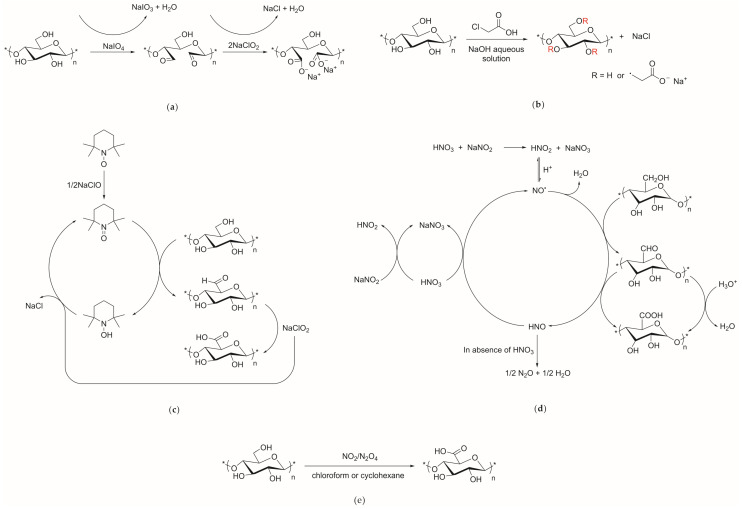
Mechanisms of cellulose carboxymethylation. (**a**) Two-step oxidation of periodate and sodium chlorite; (**b**) Carboxymethyl monochloroacetic acid; (**c**) TEMPO oxidation; (**d**) Nitric acid oxidation; (**e**) Oxidation of nitrous oxide/nitrogen dioxide system.

The carboxymethylation of cellulose has been performed for over 100 years and its main mechanisms are shown in Figure 3.

However, in the past 10 years, carboxymethylation of cellulose has been used to prepare nanocellulose. The pretreatment of cellulose by carboxymethylation to prepare nanocellulose was first proposed by Wagberg et al. In 2008, Wagberg et al. used carboxymethyl cellulose combined with high-pressure homogenization, ultrasound and other methods to prepare a novel nanocellulose. The test proved that the carboxyl groups on the surface of nanocellulose dissociated completely at pH = 10 and, with the dissociation of the carboxyl groups, a larger electrostatic repulsion force was generated between the dehydrogenation fibrils. After combining different types of electrolytes, it was found that the structure with polyelectrolytes affected the thickness of the CNFs layer. In addition, the dotted structure of carboxymethyl nanocellulose also enabled it to form a layered structure with the oppositely charged electrolyte [72]. Since then, research on the carboxymethylation of cellulose in the preparation of CNFs has gradually increased. Siró et al. prepared carboxymethylated carboxymethyl nanocellulose with a high transparency through the study of carboxymethyl cellulose and the homogenization process [73]. Aulin et al. reported that the CI of nanocellulose improved after carboxymethylation compared with enzymatic hydrolysis (type II), the particle size of carboxymethylated cellulose was lower than enzymatic hydrolysis and the product uniformity was better [74]. In addition, studies have shown that, compared with enzymatic hydrolysis, carboxymethylated nanocellulose results in stronger fibers and has better mechanical and barrier properties [75]. However, the study also showed more fiber particles in the dispersion solution of carboxymethyl nanocellulose which affected its transparency. The charged structure of carboxymethyl nanocellulose also enabled it to have better dispersion in water solvents, improved the structure stratification of the fiber network structure of the CNFs, and, compared with other kinds of cellulose, demonstrated a higher water retention rate [74,75,76].

The main disadvantage of carboxymethylation is that toxic chemicals are used in the pretreatment process, the reaction steps are complicated and cumbersome and the content of fiber fragments in the prepared CNFs is high, which reduces the product’s transparency and mechanical properties.

#### 3.2.3. Phosphorylation

The phosphorylation of nanocellulose is the pretreatment of cellulose with phosphate and nitrogen-containing organic substances, such as urea in a high oxygen state. The phosphorylation of cellulose can make the cellulose carry negative charges [77]. In addition, the heat resistance and flame retardancy of nanocellulose have also been improved because of the addition of phosphorus [78]. Maryam et al. [78,79] used (NH4)_2_HPO_4_/urea (1:4) to treat 1 wt.% sulphate wood pulp and homogenized to obtain cellulose nanofibrils with a width of approximately 3 nm. They found that the structure of the phosphorylated CNFs significantly improved the flame retardancy of cellulose due to the phosphate group in the cellulose. In the report, the nanofiber sheet was self-extinguishing after continuous application of methane flame for 3 s and did not ignite under a heat flux of 35 kW/m^2^. Maryam et al. also used layer by layer assembly of nanocellulose and impregnation with fluorosilane to obtain ultra-high-strength nanocellulose membranes with tensile strength and Young’s modulus up to 160 MPa and 9 GPa in their subsequent research. Studies have shown that the dispersion of nanocellulose can be improved by phosphate grafting, where the mechanical properties are improved compared with those before modification. However, some studies have shown that excessive phosphorylation in the experimental process will lead to the dissolution of the amorphous region of cellulose and the decrease of the DP; this causes the cellulose surface to graft with divalent phosphate groups, thereby inhibiting the formation of nanocellulose in the preparation process [80].

Phosphorylation pretreatment of cellulose can improve the mechanical strength of nanocellulose, impart flame retardancy to the fiber and introduce negative charges to the fiber surface. The pretreatment reagent is non-toxic and can be reused, so its industrialization prospects are broad.

#### 3.2.4. Cationization

The cationization of cellulose is a common cellulose pretreatment method. Similar to phosphorylation, cationization can directly introduce positive charges to the cellulose surface or rely on oxidation by an oxidizing agent and promote the fibrillation of cellulose through electrostatic action. Furthermore, the introduction of electric charge imbibes the cellulose with certain antibacterial properties [81,82,83]. Generally, the cationization of cellulose relies on the introduction of tertiary amine or quaternary ammonium groups by chemical reactions or the introduction of gas plasma [84]. Odabas et al. used isopropyl-ketone and tetrahydrofuran as fibers to study the effects of different solvents on the cationization of cellulose. When the solvent replaced 90% of the water, it greatly increased the degree of substitution and reaction efficiency. At the same time, the degree of cationization also depends on the molar ratio of the cationic reagent in the cellulose dispersion. The use of organic solvents such as tetrahydrofuran can improve the cationization. The concentration of the reagent in the dispersion can effectively maintain the CI of the cellulose and the integrity of the molecular chain [85]. After cationization, the cellulose can not only achieve better dispersion effects through electrostatic repulsion but also make the cationized hydroxyl groups become reaction sites for nucleophilic addition and provide conditions for subsequent grafting [82,86]. Since ammonium compounds are mostly antibacterial compounds with excellent performance, cationized cellulose displays certain antibacterial functions. Research by Chaker et al. showed that, after cationization, the cellulose displayed antibacterial functions when the surface charge of cellulose reached 1160 µmol/g [87,88]. Littunen et al. [82] pre-cationized pulp and epoxy propyl trimethylammonium chloride (EPTMAC) through etherification and redox grafted copolymerized trimethylammonium chloride (DMQ) to obtain cationized CNFs. The cationized nanocellulose degree of substitution (DS) reached 0.13, the ion concentration was 239 μeq/g and demonstrated effective killing of gram-positive and negative bacteria and yeast when the cytotoxicity was within a reasonable range. In addition, the DS of the cationized nanocellulose was related to the cellulose charge density and the viscosity of the cellulose dispersion [82,89].

Due to the introduction of quaternary ammonium compounds, cationized cellulose often shows excellent antibacterial properties but the cationized DS for cellulose is low, mostly below 0.5 [90]. Most of the ammonium-based cation reagents demonstrate certain cytotoxicity and pollution [82], so cationized cellulose is currently not suitable for industrial production.

#### 3.2.5. Periodate Oxidation

Contrary to the direct modification of cellulose surface hydroxyl groups, the periodate oxidation of cellulose uses periodate to selectively oxidize the hydroxyl groups at the C_2_ and C_3_ positions of cellulose in an aqueous solution and destroy the C_3_ and C_2_. The CC bond opens the ring to form an aldehyde group, the reaction activity of the generated ring-opening dialdehyde cellulose is greatly improved and hemiacetal can be formed through a nucleophilic addition reaction to promote the fibrillation of nanocellulose. However, the process of periodate oxidation is often accompanied by peroxidation and free radical depolymerization of cellulose, mainly owing to the β-elimination effect of dialdehyde cellulose in a neutral environment. In addition, periodate itself is unstable and will decompose to form free radicals as the reaction proceeds, where the decomposition effect is more obvious when exposed to light and oxygen. Therefore, in the actual reaction, the mixture is oxidized under dark and anaerobic conditions and free radical inhibitors such as isopropanol need to be added during the reaction to reduce free radical depolymerization [91]. In addition, studies by Kim and Liu et al. showed that the cleanliness of the dialdehyde cellulose obtained after periodate oxidation decreased, the flexibility increased and the content of aldehyde groups increased with increasing temperature [92,93].

Sirviö et al. [94] reported a method for producing dialdehyde cellulose ultrafine fibers by periodate oxidation. The experiment obtained cellulose fibrils with a length of 10–50 μm and a diameter of several hundred nanometers to 1 μm. In addition, experiments have found that metal salts, such as CaCl_2_ and LiCl, can act as activators to induce the oxidation of periodate and increase the aldehyde content. In addition, the experiment of Sirviö et al. also found that simultaneous grinding alongside oxidization can replace the traditional experimental conditions of high temperature and extended reaction time [94]. The experiment also confirmed that the periodate treatment greatly reduced the DP and CI of the cellulose and almost all of the crystalline areas in the cellulose were removed after the metal salt compound periodate treatment for 2 h.

The dialdehyde cellulose produced after periodate oxidation can also be further oxidized or reduced to produce 2,3–dicarboxylic cellulose and glycol cellulose. Sven et al. [95] obtained a completely independent, highly dispersible nanocellulose with a diameter of approximately 2.5 nm through the sequential oxidation of periodate-chlorite and obtained a gel with drug delivery functionality through mechanical layering. In their report, periodate and chlorite acted to control the length of the cellulose fibers and the content of the carboxyl groups, respectively. When the periodate oxidation time increased from 2 h to 4.5 h, the average length of nanocellulose changed from 375 nm to 95 nm. In addition, the degree of drug loading was directly controlled by the carboxyl content and the optimal oxidation rate was controlled at 0.74–2.00 mmol/g^−1^. Larsson et al. [96] used NaBH_4_ to reduce the dialdehyde nanocellulose fibrils oxidized by sodium periodate to obtain glycol cellulose. The glycol cellulose surrounded the cellulose fibrils to form a core-shell structure of structured nanocellulose, where a single fiber exhibited increased ductility and extremely high flexibility on the whole. Experiments showed that, when the conversion rate of C_2_–C_3_ was 27%, the tensile strength of glycol nanocellulose paper increased to 90 MPa and the breaking energy increased to 9 kJ/kg, which was 3–4 times higher than that of traditional paper. 

The periodate oxidative pretreatment of cellulose can improve the flexibility of cellulose, promote the structuring of cellulose and provide cellulose with excellent dispersibility. However, in general, the oxidation reaction time of this method is relatively long and the periodate is highly toxic and expensive, so the periodate oxidation method is limited in actual industrial production.

#### 3.2.6. Supercritical Fluid Technology

Supercritical fluid (SCF) is a special solvent for environmentally friendly and sustainable use. It is commonly used to extract cellulose, hydrolyze cellulose, impregnate cellulose or cellulose acetate and give cellulose special functions such as antibacterial, high load capacity, drug delivery and so forth [97,98]. However, cellulose has specific functions because it can be deposited directly on the polymer surface through solution or introduced additives. Meanwhile, it can destroy the firm and dense aggregate formed by cellulose fiber accumulation, increase the specific surface area and porosity of cellulose and assist to improve the efficiency of cellulase hydrolysis [98,99]. At present, this technology has been gradually applied to the pretreatment of cellulose fiber in the production of cellulose nanofibrils.

Nanta et al. [98] using supercritical carbon dioxide fluid (ScCO_2_) extract processing, processing cassava dregs cellulose fiber specific surface area from 0.75 m^2^/g increases to 12.10 m^2^/g, compared with the untreated fiber at the same time, the microstructure of treated cellulose present a more smooth and fine fibrillation network, it also makes the processing of cellulose fiber decomposition temperature increase. The supercritical fluid impregnation is used to load functional substances onto the cellulose surface in two main ways. (i) The additives dissolved in supercritical fluid are simply deposited into the cellulose matrix by reducing the system pressure;(ii) Substances with high affinity for the polymer are added to the supercritical fluid and functional groups are introduced by the chemical interaction between the additive and cellulose [100]. Milovanovic et al. [97] reported that thyme was impregnated with CA in supercritical fluid and thyme was directly introduced into THE CA after impregnation. After treatment, the CA also showed strong antibacterial properties and could continuously release thyme for up to 21 days. Compared with the traditional ionic liquid or alkali urea system, the supercritical fluid treatment eliminates the use of organic solvent and acid-base solution and the operation treatment is simpler and the product is obtained without complex washing and purification. In addition, the impregnation can be carried out in low temperature environment, can be suitable for temperature sensitive magnetic cellulose and other materials [100,101]. 

Each pretreatment process is evaluated and listed in Table 3.

## 4. CNFs Preparation

### 4.1. High Pressure Homogenization

High-pressure homogenization is the longest and most widely-use CNFs preparation method. The main principle is to repeatedly pass the cellulose dispersion through the small flow channel of the homogenization valve under high pressure conditions (>150 MPa) [105]. The dispersion produces strong peeling and shearing forces, causing the cellulose fibrillation to decrease. In addition, because of the uneven distribution of material particles and high homogenization pressure, the homogenization often causes blockage of pipelines and wear of the homogenization machine pump mouth. The equipment has a low reliability and requires complex maintenance. In addition, there are large homogenization times for preparation and high energy consumption [106]. The large size of the CNFs particles obtained from this process means that it is not often used for the preparation of CNFs alone at present [105,107,108,109,110].

### 4.2. Micro-Jet

The micro-jet method uses micro-jet technology, which is similar to the high-pressure homogenization method. The micro-jet method uses ultra-high pressure (>300 MPa) to pass the cellulose dispersion through an N-shaped or Y-shaped channel at a speed of several Mach. When ejected from a narrow valve port of 100–400 µm, cellulose is subjected to a huge shear force when flowing through the tortuous flow channel under the action of ultra-high pressure and quickly ejected from the valve port and the cellulose fiber is also affected by this process [111]. During exfoliation to the nanoscale, because of the different flow channel structure and higher pressure, the single processing efficiency of the micro-jet method is much higher than that of high-pressure homogenization [1,112]. In practical applications, cellulose can only be processed to nanoscales several times. This is the main means of the industrial production of CNFs. However, some studies have shown that the CI of CNFs will decrease under ultra-high pressure and, as the processing number increases, the creep flexibility of cellulose and the fiber network structure tend to relax [113,114].

### 4.3. Milling

The milling method mainly uses the mechanical force of a grinding disk or a planetary ball (planetary ball mill) and a fixed grooved disk to shear and rub the cellulose fiber to reduce the fiber particle size. The particle size of nanocellulose prepared by the milling method mainly depends on the distance between the grinding disks. During the preparation process, as the gap between the grinding disks continues to shrink, the cellulose fibers are continuously squeezed and sheared by the two grinding disks and the particle size of the cellulose fibers is also continuously reduced to small sizes to achieve fibrillation [115]. Compared with the high-pressure method, the production efficiency of disk milling is extremely low and it is necessary to adjust the gap between the disks for multiple grindings. When the size of the cellulose is greatly reduced, it becomes difficult to adjust the gap between the disks. At the same time, because of improper adjustment during the preparation process, the two high-speed rotating grinding disks often collide and the friction between the grinding disks introduces a large amount of metal or quartz debris, which affects the purity of nanocellulose. In addition, because there are a certain number of grooves on the surface of the grinding disk, it is easy to embed cellulose during the grinding process, resulting in an uneven cellulose scale [116]. Generally, the high-speed rotating disk mill will generate a lot of heat and cannot run continuously. However, the milling method is not prone to clogging and can process a large amount of raw materials at one time, so it is mostly used for pretreatment before enzymatic hydrolysis or homogenization of large quantities of cellulose to reduce the size of cellulose in advance [117,118,119].

### 4.4. Ultrasonic Treatment

The preparation of CNFs by ultrasonic treatment mainly relies on the joint action of a series of continuously growing and ruptured flow field bubbles (cavitation) formed by high-energy, acoustic waves in the liquid (cellulose dispersion) and sound flow conduction under the continuous action of high and low frequencies. In the preparation process, a high-energy bubble burst at ultrahigh pressure produces more than 500 atm, imposing a significant shearing force on the fiber dispersion and repeatedly applying a high frequency that is generated in the dispersion of continuous acoustic streaming [1,120]. Acoustic streaming in the conduction process results in intense mixing of the solution and further promotes fibrillation nanofibers. There is a strong relationship between the size and yield of the fiber obtained by ultrasonic treatment and ultrasonic power in ultrasonic time. Wang et al. studied the influence of various factors on the fibrillating of nanocellulose from five perspectives, such as power, time, temperature and cellulose concentration. The experiment showed that the water retention value of cellulose decreased after ultrasonic treatment and the power of the fiber decreased. The higher the temperature, the better the fibrillating of cellulose and the more effective the combination of ultrasonic and homogenization or chemical treatment was at obtaining a uniform CNFs suspension [121]. Chan et al. also prepared CNFs with a particle size of only 5–20 nm through the combination of an NaOH pretreatment to remove the non-cellulose part and high-energy ultrasound [122,123].

### 4.5. Low Temperature Pressing

The low temperature squeezing method is used to freeze the cellulose dispersion liquid swollen with water or alkali at low temperature using liquid nitrogen, form ice crystals inside the cellulose cells and then apply a strong shear and impact to the freezing system. The ice crystals formed by cellulosic cells become broken and brittle, so that the cells are broken and decomposed to release nanocellulose [119,124]. 

### 4.6. Steam Explosion

The steam explosion method uses the rapid release of high-pressure steam that penetrates into the fiber to destroy the internal structure of cellulose from the inside out. This method is mostly used in industry to purify cellulose from plants. Deepa et al. used a steam explosion treatment and showed that the α-cellulose content in banana fiber increased from 64% to 95% after treatment. It was confirmed that the high-pressure steam destroyed the non-cellulose part between the cellulose cells and the CNFs produced were similar to traditional methods. The acid treatment resulted in a higher thermal stability [124,125].

### 4.7. Electrospinning

This method requires the cellulose to be made into a solution, then a tiny jet of cellulose is ejected from the needle-shaped injection port under a strong electric field environment to form a Taylor cone and then, as the solution evaporates, nanocellulose is formed at the tail of the Taylor cone [126]. The biggest advantage of this method is that the cellulose solution can be directly adjusted via the diameter of the microjet and other parameters to obtain nanocellulose with different particle sizes and uniform sizes [72,127]. Due to the poor solubility of cellulose, the main factor limiting this method comes from the choice of solution. In response, Frey et al. developed a cellulose dissolution system based on dimethylacetamide/lithium chloride [128] and Qi et al. used the aqueous solution system of PVA/NaOH/urea (9:3:7) to prepare nanocellulose with an average diameter of 400 nm and an extremely uniform size distribution [129].

### 4.8. Solvent Method

The solvent method is similar to the electrospinning method. First, the cellulose is swollen and dispersed with a specific solvent, such as N-methyl morpholine oxide (NMMO) or *N,N–*dimethylacetamide (DMAc)/LiCl and then ultrasonically dispersed or stirred vigorously to fibrillate cellulose. In addition, adding a non-solvent phase to the dissolution system can increase the rate of cellulose fibrillation [130,131]. In this method, because of the impact of the liquid micro-flow and mechanical stirring, the cellulose easily forms a spheroid with a core-shell structure [132].

### 4.9. Ionic Liquid Method

The ionic liquid method uses organic cations or inorganic/organic anions to dissolve and separate wood cellulose. Imidazole ionic liquids are the most commonly used ionic liquids for wood cellulose processing and are commonly used for cellulose dissociation. The ionic solution systems are 1–ethyl–3–methylimidazole acetate ([EMIM][OAc]), 1–allyl–3–methylimidazole chloride ([AMIM][Cl]) and 1–Butyl–3–methylimidazole chloride ([BMIM][Cl]) [133,134,135]. After dissolution, the cellulose ion solution system changes the conditions to re-precipitate the cellulose, using electrospinning or spraying to make the cellulose rapidly precipitate and prepare the nanocellulose [133,135,136,137,138]. However, generally, the reaction of the ionic solution at room temperature is characterized by high viscosity, low solid-liquid ratio, high price and certain toxicity, so it is rarely used at present.

## 5. Surface Modification of Nanofibrils

**Figure 4:**
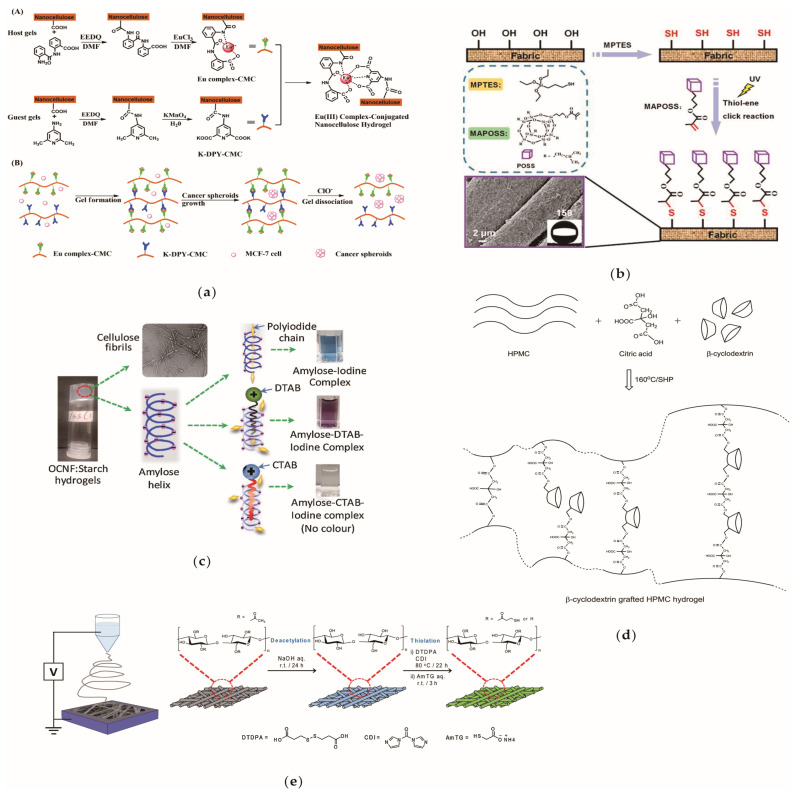
Cellulose nanofibril (CNFs) surface modification examples. (**a**) Anion luminescent nanocellulose gel [139]; (**b**) Superhydrophobic modification of cellulose fabric [140]; (**c**) Cationic modification [141]; (**d**) Cellulose was esterified and grafted with cyclodextrin [142]; (**e**) Electrospinning and sulfhydrylated cellulose [143]. Copyright 2019, 2018, 2020, 2017, 2020 respectively. Reproduced with permission from Elsevier.

Figure 4 shows the common surface modification methods of CNFs including anion luminescent nanocellulose, superhydrophobic modification of cellulose fabric and so forth. Through surface modification, additional, excellent properties can be induced in CNFs such that they can be functionally applied in various fields. At present, the surface modification of CNFs is mainly divided into two types: the surface adsorption modification of nanocellulose; and the chemical grafting of CNFs. In the chemical modification process, the modifier mainly acts on the hydroxyl groups on the 2, 3 and 6 carbon atoms and the respective reactivity of the hydroxyl groups attached to each carbon atom is different. Generally, the esterification reaction rate of the C_6_ hydroxyl group is approximately 10 times faster than that of C_2_ and C_3_ and the hydroxyl etherification reaction rate of the C_2_ position is approximately twice as fast as that of the C_3_ hydroxyl group. Regarding their dehydrogenation ability, C_2_ > C_3_ > C_6_.

### 5.1. Surface Adsorption Modification

Adsorption modifiers are mainly formed by the combination of a modifier and cellulose surface hydroxyl group to replace the hydrogen bond between cellulose. Surface adsorption modification is mainly divided into two categories: the polyelectrolyte method; and other groups involving the adsorption of points [144,145].

The polyelectrolyte method mainly combines different polyelectrolytes with opposite charges and different nanoparticles to impart certain properties to the nanoparticles and then modifies them through the adsorption of the nanoparticles and CNFs. Based on the microscopic network structure of the CNFs, polyelectrolyte adsorption is mostly used to prepare multi-layer CNFs films. The modified polyelectrolyte is often used for the thermal induction of materials and as a flocculation material device for water treatment [146,147,148]. Wagberg et al. [102] pioneered modifying cellulose using the polyelectrolyte method. They used cationic polyelectrolyte PDADMAC poly diallyl dimethylammonium chloride (PDADMAC) and polyethyleneimine (PEI) or polyallylamine hydrochloride (PAH) and then exposed to micro-fibrillated cellulose (MFC). A regular MFC and polyelectrolyte layer was formed, where the structure of the polyelectrolyte is different and smooth MFC layers with different thicknesses were obtained. Martins et al. [149] not only used PAH and PEI but also used poly diallyl dimethyl ammonium chloride (PDDA) and poly(4-styrene sulfonate) (PSS) polyelectrolyte and combined these four polyelectrolytes for use as a linking agent. The influence of different linking agents on the final properties of the composite material was studied when CNFs and silver nanoparticles were self-assembled to prepare composite materials. The results showed that all CNFs treated with polyelectrolytes exhibited strong antibacterial activity under low-concentration nutrient conditions but displayed no antibacterial activity under high nutrient concentrations.

Generally, adsorption modification involves physical adsorption without electrolyte complexation. The charge carried by CNFs enable them to be combined with better adsorption properties. Mahfoudhi et al. [150] immersed CNFs aerosols in AlCl_3_ and used NaOH to replace AlCl_3_ with Al(OH)_3_ to prepare a reusable, porous material for fluoride absorption. The report indicated that metal ions were adsorbed on the surface of CNFs, significantly improving the adsorption capacity of fluoride. The CNFs gel area, pH value (influencing CNFs charge and ion exchange) and the size of metal oxides are also key factors to improve the adsorption performance. Generally, CNFs exhibit good adsorption capacities for the same type of biomass materials. Chimphango et al. [151] used α-L-Arabinofuranosidase (AbfB) and α-D-glucuronidase (AguA) to remove the side groups of polymerized xylan using enzymes, at pH = 4.8, soaked for 16 h at 40 °C, followed by an adsorption of a soluble xylan biopolymer on the CNFs, that effectively enhanced the strength of the joints and intersections at the beginning and end of the CNFs and improved the water retention of the CNFs.

Hatton et al. [152] adsorbed the synthesized zwitterionic block copolymer of xyloglucan-*block*-Poly (sulfobetaine methacrylate) (XG-b-PSBMA_n_) on CNFs and the modified CNFs showed super high adsorption (4200%) when placed in water at 60 °C. At the same time, the fibers in the cross-section of the modified CNFs after suction filtration also showed a more regular arrangement. In addition, to improve the hydrophobicity and oil resistance of cellulose, Aulin et al. [153] used perfluorooctadecanoic acid (C_17_F_35_COOH) to dip nanofibrils. After dip coating, the surface roughness of cellulose increased and the contact angle to castor oil increased from 26° to 90°. Generally, the surface modification of CNFs by physical adsorption does not involve chemical reactions and the process is simple and convenient. However, in general, the treatment time is longer, the binding strength of the modifier and CNFs is low and the modifier molecules easily migrate from the cellulose surface.

### 5.2. Graft Modification

The graft modification of CNFs mostly operates through chemical reactions with the CNFs surface groups (–OH, –CHO, –COOH, etc.) or acting on the AGU structure of the cellulose molecular chain. The strong bond connection after graft modification of CNFs can provide CNFs with a stable structure and performance.

#### 5.2.1. Esterification

Esterification modification of nanofibrils is the most common modification method. This method mostly uses carboxylic acid or acid anhydride, for example, to react with the cellulose surface hydroxyl groups to form esters. This method is commonly used to hydrophobically modify cellulose and improve the dispersibility of CNFs to prevent their keratinization. Mulyadi et al. [154] grafted maleic anhydride styrene block copolymers onto the surface of CNFs. The results showed that the contact angle of the esterified CNFs was as high as 130° and they maintained good CI, thermal stability and the tensile strength and the elongation at break also increased by 33% and 34%, respectively. Huang et al. [155] used malic acid and 3–Aminopropyltriethoxysilane (KH550) at 110 °C to esterify CNFs using sodium bisulfate monohydrate as a catalyst. After esterification, the dispersibility of the CNFs significantly improved, the three-dimensional network structure pore size became larger and the adsorption level increased. The average CNFs diameter of the esterification group decreased but the CNFs fiber diameter increased because of the hydrolysis of KH550 and the wrapping of the CNFs. In addition, the grafting of malic acid and siloxane also caused the surface of the CNFs films to exhibit a certain roughness and the hydrophobicity of the films improved. The esterification modification of cellulose can also be used for the adsorption of heavy metal ions. Choi et al. [143] reported the use of cellulose acetate (CA) and ammonium thioglycolate (AMTG) to prepare sulfhydryl functionalized (3,3’–dithiodipropionate) by electrospinning nanocellulose and, based on the excellent chelation of thiols with metal ions, a nanocellulose film with high adsorption to heavy metal ions was prepared. Cu(II), Cd(II) and Pb(II) ions were the largest, with adsorption capacities of 49.0 mg·g^−1^, 45.9 mg·g^−1^ and 22.0 mg·g^−1^, respectively. 

The excellent gel behavior of nanocellulose makes it an excellent platform for drug delivery [156]. The esterification of cellulose makes it possible to graft a variety of drug factors and it is widely used in drug loading and sustained release [157,158,159,160]. For example, Sevinc et al. [161] reported that 3β–hydroxy–5–spirosterone (DGN) and nanocellulose were synthesized by Steglich esterification to prepare nanocellulose hydrogels that could be applied to sustained drug release and antibacterial functions. After DGN coupling, the gel system exhibited good swelling and water absorption performance at pH = 4.5–9.5 and, in an acidic environment (pH = 4), its maximum loading rate reached 95.5%. At the same time, because of the coupling effect of DGN, the mechanical properties of the hydrogel under pressure significantly improved. The increase in DGN content increased the hydrophobicity of nanocellulose and reduced the degree of gel swelling, which reduced the slow-release efficiency of the drug. Release studies also indicated that there are two release modes for sustained drug release. Ghorpade et al. [159] used citric acid, β–cyclodextrin (β–CD) and carboxymethyl cellulose for esterification and cross-linking to prepare a hydrogel film that controlled the long-term release of insoluble drugs such as ketoconazole. The incorporation improved the incompatibility between the hydrophobic drug and the loaded hydrogel and prevented the explosive release of the drug by forming an inclusion compound.

At present, the main problem facing cellulose esterification modification is reaction difficulties under mild conditions and increasing the grafting rate of the esterification modifier on the surface of cellulose to improve specific properties.

#### 5.2.2. Acylation

Similar to the esterification of cellulose, the acylation of cellulose is also modified by introducing ester groups to the cellulose and the acylation reaction is most commonly performed in organic solvents. At present, the acylation of cellulose most commonly involves carbonylation (acetylation and formylation). Generally, there are two stages for surface modification of CNFs, including acylation of the primary and secondary hydroxyl groups exposed in the nanocellulose microfiber and then separation of the surface modified cellulose by mechanical action and further reaction under the catalytic system [162,163].

Acetylation can improve the hydrophobicity and mechanical properties of cellulose and effectively prevent the keratinization of cellulose, resulting in a better dimensional stability of the CNFs [164,165,166]. The method of acetylation involves adding acetic anhydride, glacial acetic acid and sulfuric acid or perchloric acid to replace the hydroxyl groups of nanocellulose. Generally, the cellulose morphology does not change after acetylation and the drying treatment for acetylation affects the degree of acetylation of the CNFs. Zepic et al. [164] reported that spray drying improved the acetylation effect of CNFs, while freeze-drying pretreatment sped up the acetylation reaction. In addition, a higher DS (>1) causes the acylated cellulose to dissolve in water. Yetiş et al. [165] confirmed that cellulose containing high hemicellulose and lignin exhibited higher modification efficiency than cellulose without lignin and hemicellulose and could be used as a reinforcing agent or nucleation agent to improve the mechanical properties of biocomposites. In addition, to improve the dimensional stability of nanocellulose, Singh et al. [162] used propionic anhydride instead of formic anhydride and used toluene (50 mL)/pyridine (2.5 mL)/sulfuric acid (0.1 mL) to acylate the reaction medium through indirect acylation. The CNFs reaction resulted in a more stable fiber. It was reported that, under the action of pyridine, the accessibility of the cellulose surface hydroxyl groups increased and the DS increased. The performance of the prepared film also greatly improved. The contact angle was as high as 120°, the tensile strength was as high as 120 MPa, the water vapor transmission rate reduced to 101 g/(m²·day) and the light transmittance reached 80%. In addition, Bledzki et al. [167] used flax fiber for acetylation modification, studied the effect of acetylation on the structure and performance of flax and prepared modified flax fiber reinforced polypropylene composite materials. Studies have shown that, after acetylation, the surface of flax fiber is smooth and the moisture absorption performance reduces by 50% compared with that before treatment. Acetylated flax fiber was used as the reinforcing phase and added to the polypropylene substrate to prepare the composite material. The results showed that the tensile and flexural strength of the composite material first increased and then decreased with the increase in the degree of acetylation and the degree of acetylation produced the optimal mechanical properties at 18%. Mindaugas et al. [168] mechanically separated MFC from birch kraft pulp and acetylated it in toluene and then prepared a composite of polylactic acid and acetylated MFC. The results showed that, when the mass fraction of acetylated fiber was 20%, the Young’s modulus increased by approximately 70% and the tensile strength increased by approximately 60%.

Carbamylation occurs mainly through the reaction of isocyanate groups (–NCO) with hydroxyl groups (–OH) on the surface of cellulose to form carbamate bonds, thereby reducing the surface polarity of cassava residue. Huang et al. [169] used 4,4’–methylene phenyl diisocyanate (MDI) to modify cassava residue and added the modified cassava residue as a reinforcing phase to polybutylene succinate (PBS). The composite material was prepared and the hydrophobicity and mechanical properties of the composite material were studied. The results showed that, compared with the unmodified cassava residue, the contact angle of the composite material with the modified cassava residue was always greater than 100° and the tensile strength and bending strength increased by 72% and 20.89%, respectively.

Due to the toxicity of isocyanate and the need for the reaction to be performed in an organic environment, the solvent replacement process is complicated such that other modification methods are preferable.

#### 5.2.3. Silanization

Silanization of nanocellulose is a highly effective method to improve the hydrophobicity of nanocellulose. It is a common hydrophobic modification and serves as an intermediate product of subsequent grafting modification. Silanization modification usually uses polysiloxane that contains special reaction functional groups (such as sulfhydryl, double bond, fluorine, etc.), in which fluorosiloxane is used to reduce the surface energy of the cellulose membrane, while double bond and sulfhydryl provide reaction sites for subsequent modification [170]. The silanization of cellulose can be accomplished by chemical vapor deposition, plasma, sol-gel, dipping and other methods. During the treatment, the siloxane is hydrolyzed in an aqueous solution or corresponding hydroalcoholic solution and grafted onto the surface of the cellulose. It can introduce functional groups while replacing the hydrogen bonds between cellulose to improve the dispersibility of the CNFs suspensions. Musikavanhu et al. [171] prepared superhydrophobic nanocellulose paper with a water contact angle of 152.9° by grafting hexadecyl trimethoxysilane and introducing SiO_2_ by the sol-gel method to increase the surface roughness. Deng et al. used trimethoxy siloxane containing sulfhydryl groups to construct a superhydrophobic structure on the surface of nanocellulose membranes by connecting vinyl-POSS through the click reaction induced by ultraviolet light [172]. Cunha et al. used trichloromethylsilane to react with cellulose fibers to improve the hydrophobicity of cellulose fibers. The results showed that the grafted cellulose exhibited excellent hydrophobic properties and the contact angle reached 130° [173]. Yeo et al. [174] used triethoxy (3–glycidylpropyl) silane (GPS) to modify the surface of MFC and studied the silanized MFCs as a reinforcing phase to improve the mechanical properties of epoxy resin. The study showed that, compared with pure epoxy resin, the mechanical properties of the silanized composite material increased by 300% and the critical stress intensity factor (KIC) and critical strain energy release rate (GIC) increased by 1.5 times and 5.8 times, respectively. Peresin et al. [175] used a UV ozone generator to activate the surface of a CNFs film and prepared the CNFs film through APTES modification (amination) and HMDS modification (silanization) (where the DS was 0.3 and 0.7, respectively), where silanization occurred mainly in the amine modification. Chemical modification further reduced the hydrophilicity of the film (the hydrolysis contact was 70° and 60°, respectively) but the aminated CNFs film exhibited excellent oxygen barrier properties in high humidity environments (RH < 80%), while the surface monosilane completely destroyed the oxygen barrier performance. Usually, CNFs silanization requires good hydrophobicity when DS = 0.9 [176] and the accessibility of the –OH groups depends on the dispersion medium of nanocellulose. For this reason, Johansson et al. [177] took the silanization modification of nanocellulose as an example to study the influence of dispersing solvents upon exposure of cellulose surface hydroxyl groups and controlled the reaction properties of nanocellulose surface hydroxyl groups. CNFs demonstrated high purity and high hydroxyl accessibility in a medium with good dispersibility. The cellulose emulsion needs to be stabilized during the reaction to prevent CNFs from agglomeration and passivation.

#### 5.2.4. Polymer Grafting

Modification of cellulose with polymers is currently a significantly mature modification method. The modified cellulose demonstrates excellent hydrophobicity, mechanical properties, thermal stability and low degradation. Generally, the modification reaction is performed by mixing CNFs monomers and initiators to complete polymerization on the surface or grafting polymers on the surface of CNFs fibers through the action of a crosslinking agent. There are currently three polymer graft modification methods: grafting-to, grafting-from and grafting-through. As a relatively novel modification method, polymer graft modification can not only improve the dispersibility of nanocellulose, improve its hydrophobicity and improve mechanical strength but also provide nanocellulose with more novel functions, such as constructing conductive materials or semiconductor cellulose. Muiruri et al. [178] applied poly(D–lactide) (PDLA) ring opening polymerization and ε-caprolactone composite grafting of CNC and then further D - lactide polymerization and denoted as CNC–*g*–rubber–*g*–poly(D-lactide) (CNC–rD–PDLA) using CNC–rD–PDLA synthetic materials to boost the phase of the L-lactide matrix. The mechanical properties of the composites were studied and the results showed that adding the CNC–rD–PDLA increased the fracture strain of the composites by 20 times. Polymer grafting modification is limited for polymers with large molecular weight, owing to the high steric hindrance and low graft rate during graft reaction, such that intermediate weight polymers with long flexible chain segments are usually selected.

Dias et al. [179] used FeCl_3_ as the oxidant and 3–methylthiophene (3MT) as the catalyst to graft polythiophene onto the surface of the CNFs film by oxidative polymerization in a dichloromethane solution and prepared a flexible conductive film. The report indicated that the CNFs film was transformed from an insulator to a semiconductor. For semiconductor materials, the highest grafting rate in the report was 39.53% and the conductivity was 133 μS/cm. Parit et al. [180] also reported the use of polypyrrole to graft nanocellulose. In the report, the CNFs film exhibited excellent electromagnetic shielding functionality. The surface of the modified nanocellulose film was smoother and the tensile strength and wet tensile strength increased. It also exhibited a high conductivity of up to 8.4 S/cm. 

## 6. CNFs Applications

It is different from BNC in high purity, high biocompatibility and unique nanoscale structure. With high crystallinity and low length-diameter ratio of CNC, CNFs are more widely used in composite materials, antibacterial materials and food packaging and BNC is more widely used in medical scaffolds, new hydrocolloidal additives and energy storage equipment [181]. The main applications and literature references are shown in Table 4.

## 7. Results and Analysis

This review focuses on the whole process of producing cellulose nanofibrils from cellulose fibers and the various ways of preparing functionalized nanocellulose fibrils through chemical modification of nanocellulose. From the perspective of industrial production, the pretreatment of cellulose fiber is very important. The pretreatment process mainly controls the stability of the treated cellulose product quality; at present, although there are many pretreatment processes, none of them has been applied in industrial production. From the existing literature, enzymatic hydrolysis and phosphoric acid pretreatment seem to be two more suitable pretreatment processes.

In the surface modification treatment of CNFs, the modification direction is mainly aimed at improving the hydrophobic property, solubility and compatibility of CNFs in the composite substrate. In recent years, in the form of chemical grafting or physical adsorption, CNFs has also been endowed with more and more functions and gradually replaced as a new material.

In the future, with the continuous optimization of nanocellulose production and modification, industrialization and commercialization with the continuous advancement of production and the increasing demand of the people for environmentally friendly materials, nanocellulose will surely shine in more fields.

## Figures and Tables

**Table 1:** Cellulose content and fiber length of common raw materials.

Raw Material	Fiber Content (%)	Fiber Length (mm)	Fiber Diameter (µm)	Lignin Content (%)	References
Wood-Conifer	40–53	2–5	30–70	25–35	Wang et al. [28], Winandy et al. [29], Pei et al. [31], Eero et al. [32], Mishra et al. [33]
Wood-Hardwood	41–44	≈1	14–20	20–25	
Bast Fiber	65–80	–	10–25	4–20	
Herb	35–55	1–2	10–30	15–25	
Cotton Fiber	95–97	25–65	12–38	–	
Lint	90–91	10–20		<3	
Brewing Waste	16–25	–	–	11–27	

**Table 2:** Common 2,2,6,6–tetramethylpiperidine–1–oxyl radical (TEMPO) oxidation systems.

TEMPO Oxidation System	pH	Temperature Reflex (°C)	Oxidation Yield (%)	References
TEMPO/NaClO/NaBr	10	Room Temperature	75–98	Saito et al. [57]
TEMPO/NaClO/NaClO_2_	7	60	≈100	Saito et al. [65]
4–acetamide–TEMPO/NaClO/NaClO_2_	3–7	40–60	40–70	Hirota et al. [69]
4–acetamide–TEMPO Dielectric Oxidation (0.5 V)	6–8	91–98	91–98	Isogai et al. [53]
Laccase / TEMPO or Amino TEMPO	7	30	–	Jiang et al. [70], Bu et al. [71]
TEMPO/NaClO/Na_2_ SO_4_ /NaBr	10	Room Temperature	>95	Inamochi et al. [54]

**Table 3:** Cellulose pretreatment process.

Pretreatment Method	Effect on Fiber	Fiber Quality	Industrialization Prospects	References
Enzymatic Hydrolysis	DP Drops, CI Drops	High Degree of Fibrillation, no Functionalization	Excellent	Pääkkö et al. [46], Henriksson et al. [47], Nechyporchuk et al. [48]
TEMPO Oxidation	DP Drops, CI Drops	Functionalize Cellulose and Make the Surface of Cellulose Carry Charges	General	Isogai et al. [23], Tarres et al. [58], Shinoda et al. [61]
Carboxymethylation	DP Dropped Slightly, CI Basically Had no Effect	Functionalize Cellulose, which is Stronger than Enzymatically Degraded Fiber and Contains More Fiber Fragments	General	Wagberg et al. [102], Siró et al. [73], Naderi et al. [76]
Phosphorylation	DP Drops, CI Basically Has no Effect	Reinforce CNFs Fiber Strength, Impart Flame Retardancy to Fiber and Introduce Negative Charge	Excellent	Maryam et al. [79], Naderi et al. [77], Noguchi et al. [80]
Cationization	DP Drops, CI Basically Has no Effect	Improve Fiber Dispersion, can make CNFs Products have Good Antibacterial Properties or in Certain Cytotoxicity and introduce Positive Charge	Bad	Odabas et al. [85], Chaker et al. [87] Littunen et al. [82]
Periodate Oxidation	DP Drops, CI Drops	Excellent Dispersibility, Fiber Structure and Functionalization	Bad	Sven et al. [95], Larsson et al. [96], Sirviö et al. [94]
Supercritical Fluid Technology	DP Basically Has no Effect, CI Improve	Promote the Fibrillating of Fiber, Improve the Thermal Performance and Introduce Functional Groups Directly to the Surface of Cellulose	General	Stoja et al. [100], Roozbeh et al. [103], Takashi et al. [104]

**Table 4:** Functional applications of nanocellulose and related literature.

Application Field	Reference
Composite Material	Siro et al. [182], Mousa et al. [183], Mathew et al. [184]
Sensor	Dong et al. [185], Ling et al. [186], Sun et al. [187]
Food Packaging	Abdul et al. [188], Nathalie et al. [9], Li et al. [189], Azeredo et al. [190]
Food Packaging	Amjad et al. [191], Shahin et al. [192], Yang et al. [193]
Capacitor	Zhang et al. [194], Jose et al. [195], Guoet al. [196], Hou et al. [197]
Conductive Material	Xu et al. [198], Agate et al. [199]
Fireproof Materials	Costes et al. [200], Ghanadpour et al. [79], Guo et al. [201], Gebauer et al. [202]
Chemical Substance Detection	Ruiz-Palomero et al. [203]
Medical	Dumanli et al. [204], Singla et al. [205], Luzi et al. [206], Sampath et al. [207], Bhandari et al. [208]
Magnetic Material	Amiralian et al. [209], Adriano et al. [210], Olsson et al. [211]
Engineering Building	Singh et al. [212]
